# CircR2Cancer: a manually curated database of associations between circRNAs and cancers

**DOI:** 10.1093/database/baaa085

**Published:** 2020-11-11

**Authors:** Wei Lan, Mingrui Zhu, Qingfeng Chen, Baoshan Chen, Jin Liu, Min Li, Yi-Ping Phoebe Chen

**Affiliations:** School of Computer, Electronic and Information, Guangxi University, No.100 Daxue East Road, Nanning, Guangxi, 530004, China; Hunan Provincial Key Lab on Bioinformatics, School of Computer Science and Engineering, Central South University, No. 932 Lushan South Road, Changsha, Hunan, 410083, China; School of Computer, Electronic and Information, Guangxi University, No.100 Daxue East Road, Nanning, Guangxi, 530004, China; School of Computer, Electronic and Information, Guangxi University, No.100 Daxue East Road, Nanning, Guangxi, 530004, China; State Key Laboratory for Conservation and Utilization of Subtropical Agro-bioresources, Guangxi University, No.100 Daxue East Road, Nanning, Guangxi, 530004, China; State Key Laboratory for Conservation and Utilization of Subtropical Agro-bioresources, Guangxi University, No.100 Daxue East Road, Nanning, Guangxi, 530004, China; Hunan Provincial Key Lab on Bioinformatics, School of Computer Science and Engineering, Central South University, No. 932 Lushan South Road, Changsha, Hunan, 410083, China; Hunan Provincial Key Lab on Bioinformatics, School of Computer Science and Engineering, Central South University, No. 932 Lushan South Road, Changsha, Hunan, 410083, China; Department of Computer Science and Information Technology, La Trobe University Plenty Rd & Kingsbury Dr, Melbourne, Vic 3086, Australia

## Abstract

Accumulating evidences have shown that the deregulation of circRNA has close association with many human cancers. However, these experimental verified circRNA–cancer associations are not collected in any database. Here, we develop a manually curated database (circR2Cancer) that provides experimentally supported associations between circRNAs and cancers. The current version of the circR2Cancer contains 1439 associations between 1135 circRNAs and 82 cancers by extracting data from existing literatures and databases. In addition, circR2Cancer contains the information of cancer exacted from Disease Ontology and basic biological information of circRNAs from circBase. At the same time, circR2Cancer provides a simple and friendly interface for users to conveniently browse, search and download the data. It will be a useful and valuable resource for researchers to understanding the regulation mechanism of circRNA in cancers.

**Database URL:**

http://www.biobdlab.cn:8000

## Introduction

Circular RNA (circRNA) is a special class of noncoding RNA that differs from traditional linear RNAs (containing 5′ ends and 3′ ends) (1). The molecular structure of the circRNA is a closed loop, i.e. the 3′ and 5′ ends normally present in the circular RNAs are joined together. This feature confers many properties on the circular RNA, many of which have only recently been identified. In addition, circRNA is not affected by RNA exonuclease, and its expression is more stable and less prone to degradation (2). According to recent studies, circRNAs are rich in microRNA (miRNA) binding sites and act as miRNA sponges in cells. Therefore, the circRNAs can abolish the inhibition of miRNAs on their target genes and increase the expression of target genes which are known as the competitive endogenous RNA (ceRNA) mechanism (3). In recent years, with the development of high-throughput sequencing techniques, the dysregulated circRNAs have been widely detected in a wide range of cancers, including gliomas (4), esophageal cancer (5, 6), liver cancer (7, 8) and so on. In addition, circRNA is characterized by universality, tissue/cell specificity, conservation and stability, and is easily detected in human blood or saliva (9). Therefore, circRNA is becoming an ideal molecular biomarker for cancer diagnosis and treatment (10–13).

Several circRNA-related databases have been established to store circRNA-related data such as CircBase, CircNet, starBase, CircInteractome, PlantCircNet and TSCD, etc. Among them, CircBase provides users with a common and reliable circRNA data set to query and download, in which users can search for circRNAs by circRNA ID, sequence or by identifier, gene description and location (14). CircNet is the first public database to store circRNA-miRNA-gene regulatory networks and tissue-specific circRNA expression profiles which provides users with miRNA-target gene networks and expression of circRNA subtypes spectrum (15). TSCD is an online database that provides information on the characteristics and function of tissue-specific circRNA to explore the application of new RNA biomarkers in organ development (16). CircInteractome is an online knowledge base that provides users with associations between circRNAs and miRNAs or circRNAs and RNA-binding proteins (RBPs) (17). StarBase is an open source platform that provides users with a large number of high-quality RNA–RNA and protein–RNA interaction networks from CLIP-Seq (18). PlanCircNet is a repository which provides users with plant circRNA-related networks (19). In addition to the above circRNA-related databases, CIRCpedia V2 (20), Circ2Traits (21), circRNADb (22), circBank (23), CircFunBase (24), PlantcircBase (25) and the existing circRNA–disease association databases (such as circRNADisease (26), circR2Disease (27), Circ2Disease (28), Circad (29)) also provide researchers with reliable circRNA-related data. All the databases mentioned above are shown in Table 1. Although these databases can provide users with a large amount of reliable circRNA-related data. However, a database that can provide a large number of high-quality cancer-related circRNA data is still rare. Therefore, in order to meet the needs of relevant researchers, it is necessary to develop a database with a large number of reliable and cancer-related circRNAs.

**Table 1.** The circRNA-related databases.

Database	Sample	Description	URL	Reference
circBase	Homo sapiens, Mus musculus and Caenorhabditis elegans	This database contains comprehensive circRNA information and provides a variety of circRNA retrieval methods	http://www.circbase.org/	(14)
circR2Disease	Homo sapiens	This database contains 739 samples with 661 circRNA and 100 diseases	http://bioinfo.snnu.edu.cn/CircR2Disease/index.aspx	(27)
CircRNADisease	Homo sapiens	This database contains 354 samples with 330 circRNA and 48 diseases	http://cgga.org.cn:9091/circRNADisease/	(26)
circ2Disease	Homo sapiens	This database not only provides circRNA and disease relationship data but also provides miRNA and miRNA target relationship data	http://bioinformatics.zju.edu.cn/Circ2Disease/index.html	(28)
CircNet	Homo sapiens	This database not only provides basic information about circRNA, but also provides circRNA expression profile data and ceRNA regulatory network	http://www.rna-seqblog.com/circnet-a-database-of-circular-rnas-derived-from-transcriptome-sequencing-data/	(15)
starBase V2.0	Homo sapiens	A comprehensive database of miRNA information that provides not only the interactions between miRNAs and proteins but also the relationships between miRNAs and other noncoding RNAs	http://starbase.sysu.edu.cn/starbase2/index.php	(18)
CircInteractome	Homo sapiens	This database mainly includes basic information of circRNA, RBP related to circRNA and target cell sites of miRNA	https://circinteractome.nia.nih.gov/index.html	(17)
PlantCircNet	Plant	This database provides plant-related circRNA information and circRNA expression profile data as well as the circRNA-miRNA-gene regulatory network	http://bis.zju.edu.cn/plantcircnet/index.php	(19)
TSCD	Homo sapiens and mouse	This database contains information about tissue-specific circRNAs.	http://gb.whu.edu.cn/TSCD/	(16)
CIRCpedia v2	Animals	CIRCpedia V2 is a comprehensive database containing circRNA annotation information for more than 180 RNA-seq data sets from 6 different species	https://www.picb.ac.cn/rnomics/circpedia/	(20)
Circ2Traits	Homo sapiens	Circ2Traits is a comprehensive database of 105 diseases and 1951 circRNAs	http://gyanxet-beta.com/circdb/	(21)
circRNADb	Homo sapiens	CircRNADb is a specialized database that stores the basic information of 32 914 exonic circRNA	http://reprod.njmu.edu.cn/cgi-bin/circrnadb/circRNADb.php	(22)
circBank	Homo sapiens	CircBank is a specialized database for storing basic information about circRNA, which provides users with not only basic information about 140 000 circRNAs but also a large number of circRNA–miRNA interactions	http://www.circbank.cn/index.html	(23)
PlantcircBase	Plant	PlantcircBase is a comprehensive database of plant-related circRNAs. This database includes not only 77 595 circRNAs associated, but also 1335 circRNA-miRNA-mRNA regulatory networks	http://ibi.zju.edu.cn/plantcircbase/	(25)
CircFunBase	Homo sapiens and Mus musculus, etc.	This database provides not only 7000 circRNA functional instances but also a large number of circRNA–miRNA associations	http://bis.zju.edu.cn/CircFunBase/index.php	(24)
Circad	Homo sapiens and Mus musculus, etc.	This database provides a wealth of circRNA–disease-associated data	http://clingen.igib.res.in/circad/index.html	(29)

In order to address this gap, we develop a manually curated database (circR2Cancer) to provide a comprehensive and high-quality resource by retrieving published literatures and integrating available circRNA-related databases. The current version of circR2Cancer contains 1439 experimentally supported associations between 1135 circRNAs and 82 cancers. We hope that circR2Cancer can serve as useful resource for researchers to explore the regulation mechanism between circRNAs and cancers.

## Data collection and database content

**Figure 1.**
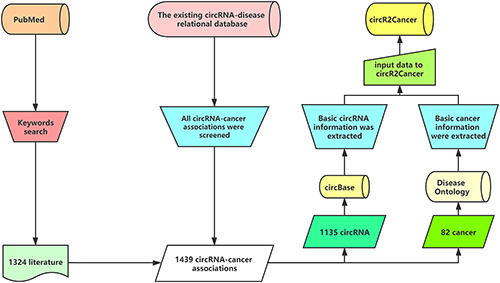


**Figure 2.**
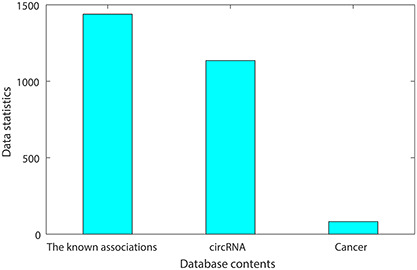


The circR2Cancer is a database for storing experimentally validated circRNA–cancer associations. The data collation process of circR2Cancer is shown in Figure 1. The data of this database are mainly derived from two parts. First, we searched the keywords ‘circRNA Cancer’ and ‘circRNA neoplasm’ on the PubMed database, and finally obtained the abstract of 1324 published articles. Then, 874 experimentally verified circRNA–cancer associations were obtained by manual method. Further, in order to enrich the circR2Cancer, we selected circRNA–cancer associations from the existing circRNA–disease associations database to enrich circR2Cancer. Finally, the circR2Cancer contains 1439 experimentally validated circRNA–cancer associations, including 1135 circRNAs and 82 cancers. The statistical information of circR2Cancer is shown in Figure 2.

In addition, circR2Cancer provides users with high-quality basic information of circRNA and cancer, respectively. The basic information of circRNA and cancer were extracted from the circBase (14) and the Disease Ontology (30), respectively. The basic information of circRNA provided by circR2Cancer including circRNA name, circRNA alias, detection method, expression pattern, gene symbol, gene coordinates, etc. At the same time, circR2Cancer provides basic information of cancers such as cancer names, DOID, definitions, synonyms, and Xrefs.

Based on above data, we designed a website interface to display these data. All data are stored and managed in the database organized by the popular open source database (MySQL). All data on our website were available to download. We used Django based on Python, Apache and MySQL systems, which is a python web framework for designing and implementing a friendly web interface for users to browse. The circR2Cancer website is freely available at http://www.biobdlab.cn:8000.

## User interface

**Figure 3.**
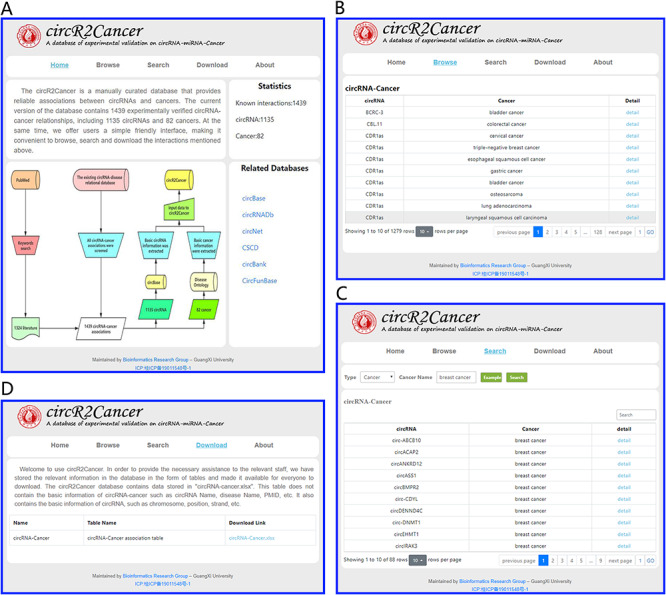


**Figure 4.**
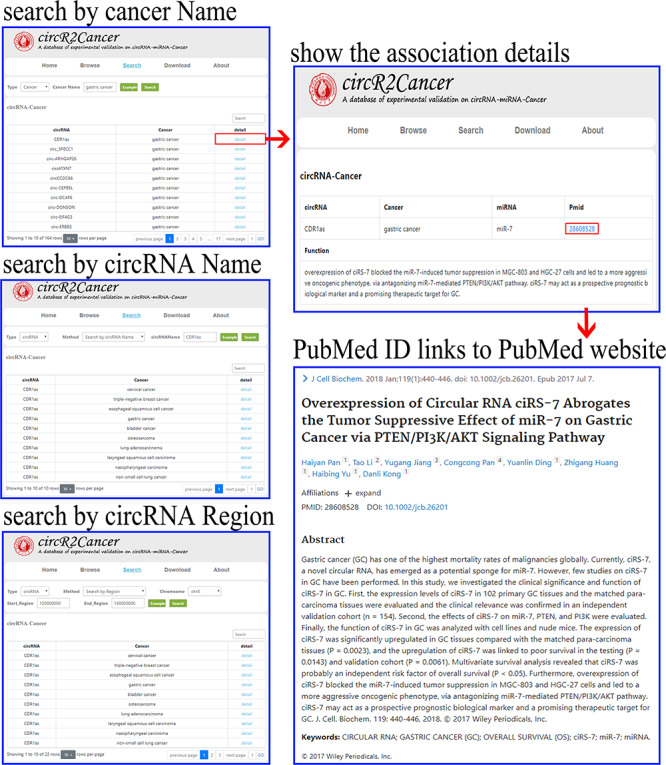


For the convenience of users, the circR2Cancer provides a simple and friendly interface to query, browse and download the data. The user interface overview of circR2Cancer is shown in Figure 3. In the ‘Home’ page, circR2Cancer provides not only an overview of the database, but also statistics information of database. Moreover, CircR2cancer also provides users with hyperlinks to other circRNA-related databases. In the ‘Browse’ interface, circR2Cancer displays the circRNA–cancer association to the user. Furthermore, the user can click on ‘Detail’ to view the details. In order to facilitate users to query the experimentally verified circRNA–cancer associations, the circR2Cancer database provides users with circRNA-based search methods and cancer-based search methods in the ‘Search’ page. The cancer-based search method requires the user to enter a specific cancer name, and then circR2Cancer displays the circRNA–cancer associations based on the cancer name entered by the user. Moreover, circR2Cancer provides two different circRNA-based search methods. (i) Search for the experimentally verified circRNA–cancer association based on the circRNA name; and (ii) search for known associations based on coordinate. The search method based on coordinate requires the user to select the chromosome of circRNA, and then inputs the start and end positions of circRNA. Finally, circR2Cancer displays the corresponding circRNA–cancer associations according to the user’s selection and input. The specific operations of the three search methods provided by circR2Cancer are shown in Figure 4.

## Discussion and conclusions

Increasing studies have shown that circRNAs are related to various human cancers such as gastric cancer (31–34), hepatocellular carcinoma (35–37), bladder carcinoma (38–41) and so on. Furthermore, recent researches indicate that circRNAs can be considered as biomarkers for cancer diagnosis, treatment and prognosis (20). Thus, in order to facilitate future research of the regulation mechanism of circRNA in cancer, we develop a comprehensive database which provides experimentally confirmed associations between circRNAs and cancers. Specifically, the circR2Cacner contains 1439 experimentally validated circRNA–cancer associations including 1135 circRNAs and 82 cancers. Meanwhile, we also provide users with a friendly and easy-to-use web interface that allows users to search, browse and download circRNA–cancer associations.

## Future extensions

With the increasing experimentally validated circRNA–cancer associations, the circR2cancer database will be updated regularly. Besides, we will add RNA-seq, function information of circRNAs and circRNA-binding protein associations. In the meantime, we will develop new algorithms and tools for analyzing circRNA–cancer associations in the future.

## onflict of interest

We declare no competing interest.

## Availability

The circR2Cancer database website is freely available at http://www.biobdlab.cn:8000.
